# Profound Perturbation in the Metabolome of a Canine Obesity and Metabolic Disorder Model

**DOI:** 10.3389/fendo.2022.849060

**Published:** 2022-04-19

**Authors:** Weiyi Qu, Ze Chen, Xing Hu, Toujun Zou, Yongping Huang, Yanyan Zhang, Yufeng Hu, Song Tian, Juan Wan, Rufang Liao, Lan Bai, Jinhua Xue, Yi Ding, Manli Hu, Xiao-Jing Zhang, Xin Zhang, Jingjing Zhao, Xu Cheng, Zhi-Gang She, Hongliang Li

**Affiliations:** ^1^ Department of Cardiology, Renmin Hospital of Wuhan University, Wuhan, China; ^2^ Institute of Model Animal, Wuhan University, Wuhan, China; ^3^ Department of Cardiology, Zhongnan Hospital of Wuhan University, Wuhan, China; ^4^ Department of Urology, First Affiliated Hospital of Gannan Medical University, Ganzhou, China; ^5^ Gannan Innovation and Translational Medicine Research Institute, Gannan Medical University, Ganzhou, China; ^6^ College of Life Sciences, Wuhan University, Wuhan, China; ^7^ College of Veterinary Medicine, Huazhong Agricultural University, Wuhan, China; ^8^ School of Basic Medical Science, Wuhan University, Wuhan, China; ^9^ Department of Radiology, Zhongnan Hospital of Wuhan University, Wuhan, China; ^10^ Department of Pathophysiology, School of Basic Medical Sciences, Gannan Medical University, Ganzhou, China; ^11^ Department of Cardiology, Tongren Hospital of Wuhan University and Wuhan Third Hospital, Wuhan, China

**Keywords:** metabolomics, obesity, metabolic disorder, high-fat diet, metabolic profiles, energy intake, biomarkers

## Abstract

Canine models are increasingly being used in metabolic studies due to their physiological similarity with humans. The present study aimed to identify changes in metabolic pathways and biomarkers with potential clinical utility in a canine model of obesity and metabolic disorders induced by a high-fat diet (HFD). Eighteen male beagles were included in this study, 9 of which were fed a HFD for 24 weeks, and the remaining 9 were fed normal chow (NC) during the same period. Plasma and urine samples were collected at weeks 12 and 24 for untargeted metabolomic analysis. Dogs fed a HFD showed a gradual body weight increase during the feeding period and had hyperlipidemia, increased leukocyte counts, and impaired insulin sensitivity at week 24. Plasma and urine metabonomics analysis displayed clear separations between the HFD-fed and NC-fed dogs. A total of 263 plasma metabolites varied between the two groups, including stearidonic acid, linolenic acid, carnitine, long-chain ceramide, 3-methylxanthine, and theophylline, which are mainly engaged in fatty acid metabolism, sphingolipid metabolism, and caffeine metabolism. A total of 132 urine metabolites related to HFD-induced obesity and metabolic disorders were identified, including 3-methylxanthine, theophylline, pyridoxal 5’-phosphate, and harmine, which participate in pathways such as caffeine metabolism and vitamin digestion and absorption. Eight metabolites with increased abundance (e.g., 3-methylxanthine, theophylline, and harmine) and 4 metabolites with decreased abundance (e.g., trigonelline) in both the plasma and urine of the HFD-fed dogs were identified. In conclusion, the metabolomic analysis revealed molecular events underlying a canine HFD model and identified several metabolites as potential targets for the prevention and treatment of obesity-related metabolic disorders.

## 1 Introduction

Obesity is a growing global concern due to its skyrocketing prevalence and the associated cluster of cardiometabolic diseases that decrease quality of life and expectancy (1). Convincing evidence indicates that obesity significantly increases the risk for conditions such as type 2 diabetes, cardiovascular diseases, and certain cancers (2). It has been reported that the global prevalence of obesity has nearly tripled over the last four decades, with 13% of adults worldwide being obese and 39% being overweight (1, 2). The management of obesity and its related metabolic disorders is a critical challenge for health care providers (3). Lifestyle modifications such as dietary interventions and physical activity are the mainstay of obesity management; however, they often fail to achieve sustained weight loss in daily practice (4). Currently, few anti-obesity drugs have been shown to be safe and effective across diverse obese populations (5). These findings call for an urgent need to better elucidate the underlying molecular mechanisms and to identify biomarkers with potential clinical utilities for the prevention and treatment of obesity and its associated metabolic disorders.

Rodent models of high-fat diet (HFD)-induced obesity have been extensively applied to study human metabolic disorders (6–9). However, these rodent models are largely limited by their capacity to accurately mimic the metabolic aspects of human diseases (10). Animals with large sizes, such as dogs, have more physiological similarity with humans than rodents, and dogs have long been used in conducting metabolic studies (11–13). Many endocrine and metabolic disorders, such as type 2 diabetes, are common to humans and dogs (11). Indeed, both species develop obesity and insulin resistance after excessive carbohydrate or fat intake (14–16). Moreover, humans and dogs share a larger degree of genetic homology than humans and rodents (11). Thus, the results derived from canine models can be better extrapolated to humans and further facilitate clinical translation.

Metabolomics approaches have been increasingly applied to interrogate molecular and metabolic alterations in metabolic diseases and are considered one of the signposts of clinical translation (17, 18). The characterization of the metabolites that are associated with metabolic disorders may provide insights into the molecular mechanisms and define biomarkers to improve patient diagnosis, prognosis, and treatment efficacy (19, 20). Although several studies have revealed metabolic signatures associated with obesity in humans and rodent models, there are few metabolomic profiling studies on canine obesity to date (21–23). Forster et al. compared plasma, fecal, and urine nontargeted metabolomes from 66 clinically healthy adult companion dogs of 22 different breeds according to their body condition score (BCS) and found that the levels of several plasma phospholipid moieties were notably different between the weight phenotypes (21). Another study by Soder et al. investigated alterations in the serum and urine metabolome in 12 lean and 16 spontaneously overweight dogs and reported lower plasma carnitine concentrations and lower postprandial urine taurine concentrations in overweight dogs than in lean dogs (22, 23). However, these studies have been limited by focusing on a relatively small number of metabolites or obesity phenotypes, and the results are confounded by several important factors, such as age, breed, sex, and background diet. Longitudinal canine metabolomic alterations in response to an HFD in a laboratory setting have not been investigated.

Here, we used a canine model of obesity and metabolic disorders induced by HFD, with longitudinal measurements of anthropomorphic data, complete blood counts, serum biochemistry, imaging examination, and plasma and urine metabolome, in an effort to obtain a comprehensive picture of the pathophysiology and metabolic phenotype of canine obesity and metabolic disorders and to discover biomarkers with potential clinical utilities.

## 2 Materials and Methods

### 2.1 Animals and Experimental Design

Experiments were performed with 1- to 2-year-old male beagles weighing 8.0–10.5 kg in a controlled standard environment (12-hour light/dark cycle, temperature 23.5 ± 1°C, relative humidity 60 ± 10%) with *ad libitum* access to water. All animal experiments complied with the Guide for the Care and Use of Laboratory Animals released by the National Academy of Sciences and the National Institutes of Health. Abuse and punishment were excluded from our study.

**Figure 1:**
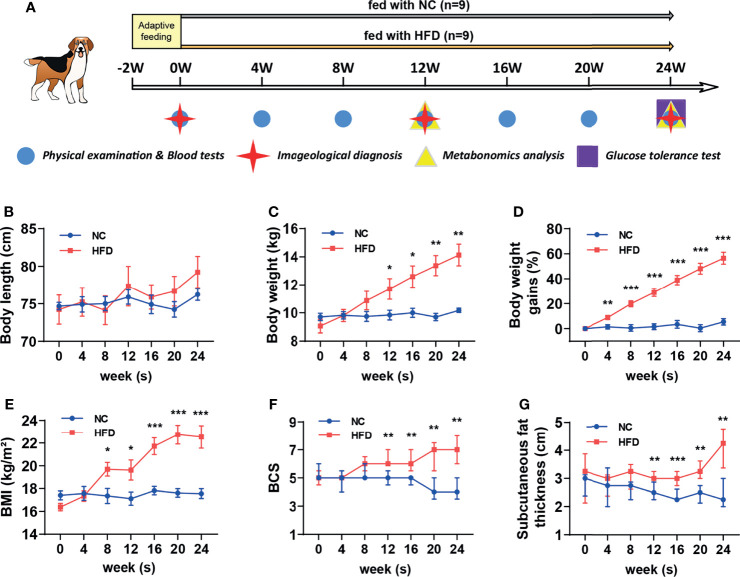
Comparison of body features between dogs with high-fat diet-induced obesity and metabolic disorder and dogs fed with normal chow. **(A)** Schematic diagram of feeding, grouping and examining regimen in this study. After 2 weeks of adaptation, a preliminary examination was carried out, and then 18 beagles were randomly assigned to receive normal chow (NC) or a customized high-fat diet (HFD) for 24 weeks. **(B–E)** Body length, weight, percentage of body weight gain compared to the baseline and BMI from NC and HFD beagles. n = 9 per group. Data are expressed as the mean ± s.e.m. Significance levels *P < 0.05, **P < 0.01, ***P < 0.001. **(F, G)** BCS and subcutaneous fat thickness from NC and HFD beagles. n = 9 per group. Data are expressed as the median with interquartile range. Significance levels **P < 0.01, ***P < 0.001.

After two weeks of adaptation, the beagles received a complete physical and nutritional examination, which assessed the body mass index (BMI) and BCS, and underwent imaging and blood tests. Eighteen beagles whose physical and laboratory test results met the healthy reference range were randomly grouped into a normal chow group (NC, n = 9) and a metabolic overload group simulated by a high-fat diet (HFD, n = 9). The NC dogs were fed a basal maintenance diet (3615 kcal/kg), and the HFD dogs were fed a customized diet rich in lipids (4832 kcal/kg). Both the basic and customized feed were ordered from Jiangsu Xietong Bioengineering Corporation (Nanjing, China). The formula and nutrient element composition of the diets are described in **Table 1**. During the study period, physical examinations and blood sampling were performed every 4 weeks. All biological samples were stored at −80°C until analytical processes were performed. After determining fasting blood glucose levels at the 24th week, a modified intravenous glucose tolerance test (IVGTT) was performed. This frequently sampled glucose tolerance test with the administration of exogenous insulin is an approach to simultaneously examine glucose tolerance and insulin resistance in HFD-induced obese dogs, and to mimic the complex situation of metabolic disorder. Specifically, each animal was given intravenous glucose according to 0.3 g/kg body weight, and then intravenous blood glucose was detected every 2 min from 4 to 18 min. Then, 0.02 U/kg body weight insulin was injected intravenously at 20 min, and blood glucose levels were examined repeatedly at 21/23/25/27/30/40/50/60/90/120/180 min. The entire animal experimental procedure is illustrated in **Figure 1A**.

**Table 1:** Ingredient and macronutrient composition of the diets fed to dogs.

	NC	HFD
**Basic maintenance feed (%)**	100.0	46.5
**Cocoa butter (%)**	0.0	12.0
**Lard (%)**	0.0	15.0
**Sucrose (%)**	0.0	10.0
**Casein (%)**	0.0	13.0
**Mineral premix (%)**	0.0	3.5
**Total energy (kcal/kg)**	3615	4832
**Carbohydrate energy ratio (%)**	59.2	26.9
**Protein energy ratio (%)**	27.1	19.8
**Fat energy ratio (%)**	13.7	53.3
**Carbohydrate quality ratio (%)**	53.5	32.5
**Protein quality ratio (%)**	24.5	24.0
**Fat quality ratio (%)**	5.5	28.6
**Fiber quality ratio (%)**	3.0	1.4
**Ash quality ratio (%)**	6.0	4.5
**Mineral quality ratio (%)**	1.8	1.7

NC group was merely fed a basic maintenance fodder. HFD group was fed a customized fodder that adds more lipin, sucrose and other ingredients to the basic maintenance feed. HFD group intakes about one-third more energy for the same weight of feed than NC group, and more than half of total energy is provided by fat. Both kinds of feed were ordered from one same company.

### 2.2 Imageological Examination and Abdominal Fat Measurement

At the 12th and 24th weeks, two radiologists with 5 and 3 years of experience, respectively, from Zhongnan Hospital of Wuhan University examined the distribution mode and relevant parameters of the subcutaneous and visceral fat of beagles through magnetic resonance imaging (MRI) (MAGNETOM Prisma 3.0T, Siemens Medical Systems, Germany) and computed tomography (CT) (SOMATOM Definition, Siemens Medical Solution, Germany) imaging. The number of animals per group for the imaging study were predetermined with an α value of 0.05 and a 1-β value of 0.8 on the basis of preliminary experiments using similar methods. The animals were anesthetized by the intramuscular injection of a 0.1 mL/kg body weight tiletamine/zolazepam hydrochloride mixture (Zoletil^®^50, VIRBAC, France). During the entire MRI process, 2–3 L/min air containing 1–2% isoflurane was inhaled through a mask to maintain the anesthesia of the animals.

After the radiologist with five years of experience selected a proper axial image between the level of the L3–L4 vertebrae transverse processes, visceral adipose tissue (VAT) and subcutaneous adipose tissue (SAT) area were measured with a workstation (Syngo. Via, Siemens Healthcare, Germany) according to the methodology previously described by Faria et al. (24). Then, the VAT and SAT areas were summed to obtain the total adipose tissue (TAT) area. The density range of adipose tissue is defined as −150 to −50 Hounsfield units, and muscle density is defined by 0 to 200 Hounsfield units. Adipose tissue was classified as VAT or SAT according to the density of intersected muscle. Two radiologists carried out MRI or CT measurements independently and were unaware of any information of animals other than their serial number.

### 2.3 Blood Sampling and Serological Analyses

After overnight-fasted animals completed the physical examination every four weeks, a blood collection needle was inserted into the distal cephalic vein to sample venous blood. Serum tubes were coagulated at room temperature for 30 min and then centrifuged at 1500 g for 10 min. Plasma tubes were centrifuged as described above directly after sampling. The serum and plasma were transferred to polypropylene EP tubes and immediately quick-frozen in liquid nitrogen. Another small amount of whole blood was collected, and the needle was withdrawn. The small amount of blood remaining on the needle tip was used to contact the high-sensitivity microdetection test paper which was matched with a glucometer (Accu-Chek Active, Roche Diagnostics). After the blood drop was absorbed, fasting blood glucose was measured by immediately placing the test paper into the glucometer. Whole blood and serum samples were submitted to routine blood tests (see **Table 2** for the results) and serum biochemical analysis (including liver and kidney function, lactate dehydrogenase, creatine kinase, and blood lipids) in the Clinical Laboratory of Wuhan Third Hospital.

**Table 2:** Blood Routine Examination of NC and HFD-fed Dogs.

Analyte	0W			12W			24W		
	NC	HFD	*P* value	NC	HFD	*P* value	NC	HFD	*P* value
	n = 9	n = 9		n = 9	n = 9		n = 9	n = 9	
**WBC (10^9^/L)**	9.8 ± 0.7	10.0 ± 1.2	.861	9.1 ± 0.5	11.4 ± 0.2	**.001**	11.3 ± 0.8	15.4 ± 0.9	**.003**
**Lymphocytes (10^9^/L)**	2.0 ± 0.2	1.7 ± 0.2	.251	2.8 ± 0.2	2.9 ± 0.1	.835	2.3 ± 0.2	2.9 ± 0.2	**.046**
**Monocytes (10^9^/L)**	0.56 (0.28)	0.45 (0.15)	.436	0.60 (0.20)	0.60 (0.10)	.340	0.48 (0.27)	0.81 (0.07)	**.011**
**Neutrophils (10^9^/L)**	6.2 (1.7)	7.0 (4.1)	.730	5.3 (1.3)	7.5 (0.7)	**.006**	9.0 (2.1)	10.4 (3.3)	**.011**
**Eosinophils (%)**	1.1 (1.1)	1.1 (1.4)	.546	0.6 (0.4)	0.7 (0.2)	.436	0.8 (0.5)	1.6 (2.1)	.489
**RBC (10¹²/L)**	6.9 (0.6)	6.6 (0.3)	.161	7.1 (1.3)	6.5 (0.5)	.136	6.8 (1.1)	7.0 (0.4)	.730
**HGB (g/L)**	148 (16)	147 (11)	.489	150 (21)	137 (14)	.113	143 (13)	149 (13)	.436
**HCT (%)**	42.0 (5.5)	42.1 (2.9)	.730	43.6 (5.8)	39.7 (3.8)	.297	45.1 (5.3)	46.2 (3.5)	.730
**MCV (fL)**	61.2 ± 0.5	61.9 ± 0.5	.312	62.3 ± 0.5	62.7 ± 0.4	.579	66.4 ± 0.9	66.7 ± 0.8	.851
**MCH (pg)**	21.2 ± 0.2	21.4 ± 0.2	.540	21.2 ± 0.2	21.5 ± 0.2	.351	21.0 ± 0.2	21.0 ± 0.2	.871
**MCHC (g/L)**	348 (4)	343 (6)	.340	339 (12)	345 (8)	.340	316 (8)	317 (14)	.999
**RDW (%)**	13.4 (0.8)	12.7 (0.5)	.113	13.7 (0.7)	13.7 (0.6)	.863	16.0 (0.6)	16.8 (0.4)	**.006**
**Platelets (10^9^/L)**	244 (93)	236 (62)	.436	316 (95)	379 (76)	.340	216 (140)	160 (174)	.999
**MPV (fL)**	7.5 (0.4)	7.6 (0.6)	.387	6.5 (0.9)	6.4 (0.7)	.863	10.8 (1.4)	10.3 (1.7)	.222
**PDW (%)**	15.0 (1.1)	15.1 (0.6)	.796	16.2 (0.5)	16.3 (1.0)	.666	17.9 (2.3)	16.9 (2.7)	.222
**PCT (%)**	0.18 (0.05)	0.19 (0.05)	.666	0.21 (0.05)	0.23 (0.05)	.222	0.23 (0.18)	0.17 (0.15)	.546

WBC, white blood cell; RBC, red blood cell; HGB, hemoglobin; HCT, hematocrit; MCV, mean corpuscular volume; MCH, mean corpuscular hemoglobin; MCHC, mean corpuscular hemoglobin concentration; RDW, red cell distribution width; MPV, mean platelet volume; PDW, platelet distribution width; PCT, plateletocrit. Data with normal distributions are reported using mean ± s.e.m; data with skewed distributions are reported using median (interquartile range). Bold are significant p-values (P < 0.05).

### 2.4 Untargeted Metabolomics

After slowly thawing the plasma and urine samples at 4°C, 100 μL of each sample was vortexed and mixed with 400 μL precooled methanol/acetonitrile/aqueous solution (2:2:1, v/v). Then, the samples were ultrasonicated at low temperature for 30 min, rested at −20°C for 10 min, and centrifuged for 15 min (14000 g, 4°C) to remove proteins from the sample. The obtained supernatant was dried with nitrogen in a vacuum centrifuge. For untargeted metabolomics analysis, the dried sample was redissolved in 100 μL acetonitrile aqueous solution (1:1, v/v) and vortically mixed. The mixture was centrifuged again at 14000 g for 15 minutes at 4°C, and the supernatant was collected for analysis.

In this study, an ultrahigh-performance liquid chromatography system (Agilent 1290 Infinity, Agilent, USA) tandem with a quadrupole time-of-flight mass spectrometer (AB Triple TOF 6600, AB Sciex, Canada) in Shanghai Applied Protein Technology (Shanghai, China) was used to perform nontargeted metabolomics. Metabolites of plasma samples collected in the 12th week, as well as plasma and urine samples collected in the 24th week, were separated on a chromatographic column (ACQUITY UPLC BEH Amide, 1.7 μm, 2.1 mm × 100 mm column) under the optimized conditions (column temperature 25°C; flow rate 0.5 mL/min; injection volume 2 μL) to achieve chromatographic separation according to the following gradient program. The elution procedure (mobile phase A: water with 25 mM ammonium acetate and 25 mM ammonia; phase B: acetonitrile) was as follows: 0–0.5 min, 95% B; 0.5–7 min, B linearly changed to 65%; 7–8 min, B linearly changed to 40%; 8–9 min, B maintained at 40%; 9–9.1 min, B linearly changed to 95%; and 9.1–12 min, B maintained 95%. Samples were placed in the autosampler at 4°C during the whole process. Continuous sample analysis was performed in random order, and quality control samples were inserted in the queue to monitor and evaluate the stability of the system. After chromatographic separation, mass spectrometry was applied, and electrospray ionization (ion source temperature: 600°C) was performed for positive and negative ion mode detection (ion spray voltage floating: ± 5500 V). The m/z range in MS-only acquisition mode was set from 60 to 1000 Da, and its scanning accumulation time was 0.20 s/spectrum. The m/z range in auto MS/MS acquisition mode was set from 25 to 1000 Da, with an accumulation time of 0.05 s/spectra. The product ion scan adopts information-dependent acquisition with peak intensity screening mode selected. The declustering potential is ± 60 V (corresponding to the positive and negative modes, respectively), and the collision energy is 35 ± 15 eV. Isotopes within 4 Da were dynamically excluded, and 10 candidate ions were collected for each cycle.

The original data in Wiff format were converted into MzXML files by ProteoWizard and then imported into XCMS software to obtain available data, followed by peak alignment, retention time correction, and peak area extraction. As previously described (25), metabolite identification was performed based on the comparison between the data extracted by XCMS with an in-house database established with available authentic standards and a recursive algorithm.

### 2.5 Multivariate Data Analysis

After sum-normalization, the ropls R package (version 1.26.0, http://www.bioconductor.org/) was used to implement a multivariate model, including Pareto-scaled principal component analysis (PCA) and orthogonal partial least squares discriminant analysis (OPLS-DA), consequently reducing the dimension of the matrix and single out the difference variables. Herefrom, we obtained 7-fold cross-validated Q2 and R2Y and the permutation-tested probabilities of Q2 and R2Y and calculated the variable importance for the projection (VIP) in the OPLS-DA model. The VIP value of each variable indicates the contribution of a specific metabolite to the classification. A VIP value > 1 and a corresponding *P* value < 0.05 were adopted to screen the metabolites with significant changes.

### 2.6 Analysis of Differential Metabolites

Differential metabolites screened by positive and negative ion modes in the preceding process were combined and analyzed using an in-house R script. The resulting metabolites were proofread with HMDB version 5.0 (https://hmdb.ca/) to identify whether their origin was endogenous or exogenous. Then, each Kyoto Encyclopedia of Genes and Genomes (KEGG) pathway term was designated as a gene set and used to annotate metabolites. In KEGG pathway enrichment analysis, whether the metabolites belonging to a certain pathway appear more frequently than would be anticipated by random chance was assessed to determine the metabolic and signal transduction pathways that were markedly affected. In the bubble diagram, a z score method was utilized to normalize the levels of various metabolites by the formula y = (real value−mean of all samples)/variance of all samples. A differential abundance (DA) score was adopted to capture the overall trend of all metabolites in a pathway. A Benjamini-Hochberg corrected Mann-Whitney U nonparametric test was applied in this study to calculate the differential abundance score of all metabolites in a certain pathway. The increase/decrease in metabolite abundance was defined as a positive/negative DA score. Specifically, a score of 1 indicates an increase in the abundance of all metabolites in the pathway, while a DA score of −1 indicates a decrease in the abundance of all metabolites.

### 2.7 Statistical Analysis

Statistical analysis was implemented *via* SPSS Statistics 20.0 (IBM Corporation, Chicago, USA). Two-tailed Student’s t test was used to compare the significance of the difference between two normally distributed independent samples, and the Mann-Whitney U nonparametric test was applied to compare two skewed-distributed independent samples. For data that met the normality assumption, Pearson’s correlation analysis was performed to investigate the correlation between beagle phenotypes in physical and blood examinations and metabolite levels in plasma and urine. Venn diagrams were generated using the online tool Venny 2.1.0 (https://bioinfogp.cnb.csic.es/tools/venny/) to demonstrate the intersection of metabolites. Significance levels (*P < 0.05, **P < 0.01, ***P < 0.001) and the number of biological replicates are formulated in each figure and figure legend. The results are presented in GraphPad Prism 9 software (GraphPad Software, San Diego, USA).

## 3 Results

### 3.1 HFD Induced Significant Obesity in Dogs

As shown in **Figure 1A**, eighteen male beagles were included in the present study, 9 of which were fed a HFD for 24 weeks, and the remaining 9 were fed normal chow (NC) during the same period. Physical examinations and blood tests were conducted every 4 weeks. Imageological examinations were conducted at weeks 0, 12, and 24. Plasma and urine samples were collected at weeks 12 and 24 for untargeted metabolomic analysis. An IVGTT was performed at week 24 to estimate whether the insulin sensitivity is impaired or not. The body length did not differ significantly between the NC and HFD groups (**Figure 1B**). HFD induced a continuous weight gain during the feeding period. At week 24, a HFD resulted in approximately 60% weight gain (**Figures 1C, D**). Body mass index (BMI), body condition score (BCS), and subcutaneous fat thickness were significantly increased by HFD (**Figures 1E–G**).

**Figure 2:**
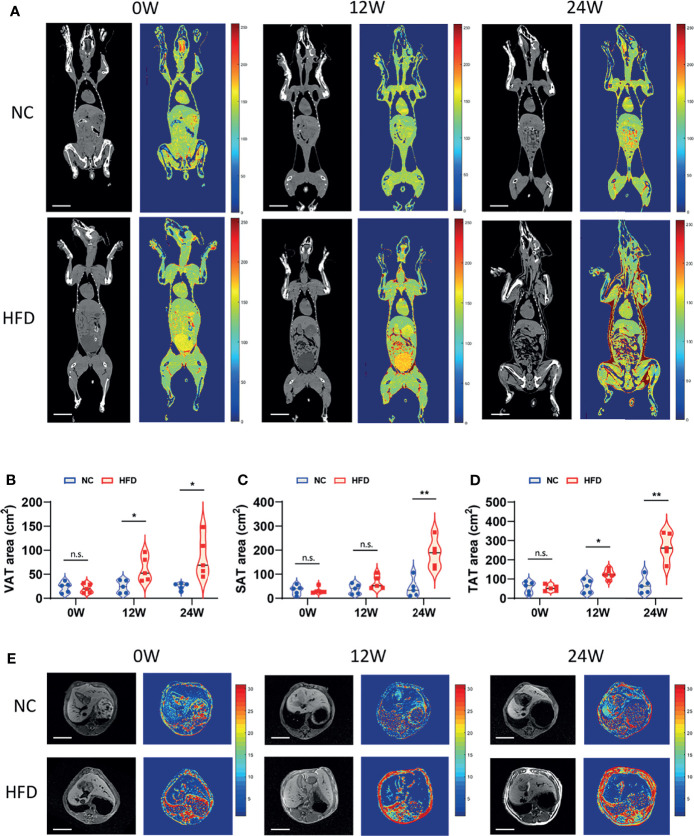
Imageological diagnosis revealed the effects of long-term intake of HFD on the body fat content and distribution of animals. **(A)** CT image of the coronal plane of NC and HFD dogs at 0, 12 and 24 weeks. Grayscale and pseudocolor images suggested that abdominal fat content increased after HFD feeding for 12 weeks, while both subcutaneous fat and visceral fat had pronounced accumulation by 24 weeks. Scale bar, 10 cm. **(B–D)** The area of visceral adipose tissue (VAT) and subcutaneous adipose tissue (SAT) measured separately based on CT imaging and the total adipose tissue (TAT) area obtained by the summation. n = 5 per group. Data are expressed in the form of violin plot. n.s. represents not statistically significant. Significance levels *P < 0.05, **P < 0.01. **(E)** Cross-sectional MRI at 0, 12 and 24 weeks shows clear changes in abdominal adipose tissue. Scale bar, 5 cm. In **(A, E)**, all the images shown in each row (NC or HFD) were obtained from the same individual dog.

We conducted CT scans and MRI to further depict the effects of HFD on the content and distribution of body fat. An obviously higher content of visceral fat and subcutaneous fat was observed in HFD-fed dogs than in NC-fed dogs based on CT images at week 24 (**Figure 2A**). In line with this observation, quantitative analysis showed a significantly higher area of visceral adipose tissue (VAT), subcutaneous adipose tissue (SAT), and total adipose tissue (TAT) in the HFD group than in the NC group at week 24 (**Figures 2B–D**). MRI showed consistent results with the CT imaging examinations (**Figure 2E**).

### 3.2 HFD Led to Increased Inflammatory Cell Counts, Dyslipidemia, and Insulin Resistance in Dogs

**Figure 3:**
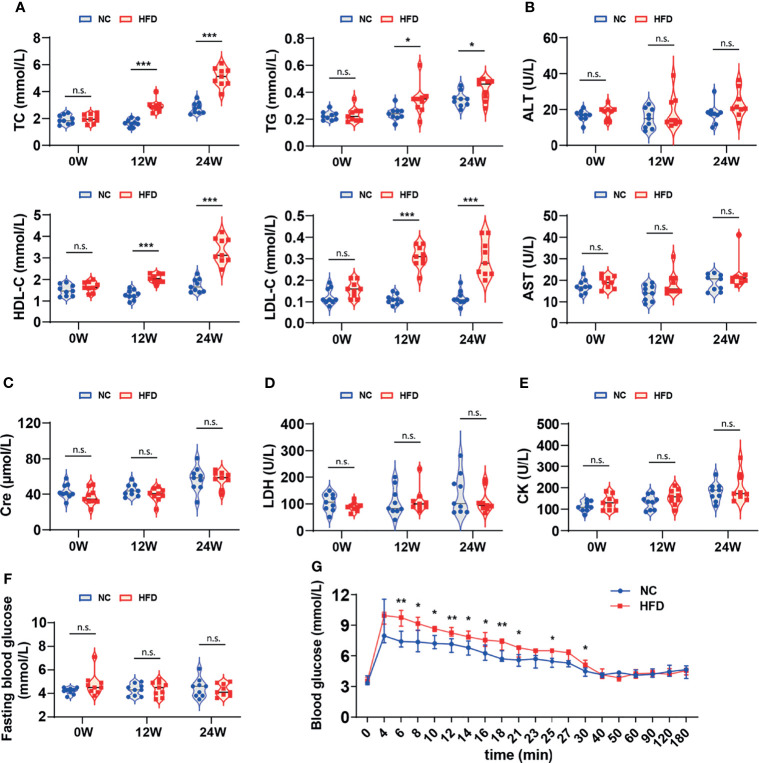
Differences in serum biochemistry and glucose tolerance between HFD-induced metabolic disorder and NC dogs. **(A–F)** The levels of serum biochemical markers reflected the blood lipids, hepatorenal and cardiac function of dogs. Fasting blood glucose was measured in dogs that fasted overnight. n = 9 per group. Data are expressed in the form of violin plot. n.s. represents not statistically significant. Significance levels *P < 0.05, ***P < 0.001. **(G)** In the modified intravenous glucose tolerance test, the blood glucose level of the HFD group was dramatically higher than that of the NC group during most of the first 30 minutes, indicating the presence of insulin resistance. n = 6 in NC group, and n = 8 in HFD group. Data are expressed as the median with interquartile range. Significance levels *P < 0.05, **P < 0.01.

**Table 2** shows the levels of 16 whole blood parameters for the NC and HFD groups at weeks 0, 12, and 24. The most striking alterations were observed in white blood cell (WBC) counts, lymphocyte counts, monocyte counts, and neutrophil counts at week 24, with significantly higher levels in the HFD group, indicating a state of inflammation and immune activation. In addition, the level of red cell distribution width (RDW) was significantly increased in the HFD group at week 24. Of note, HFD induced marked dyslipidemia at weeks 12 and 24, manifested as significantly higher levels of TC, TG, LDL-C, and HDL-C (**Figure 3A**). Other parameters, including ALT, AST, creatinine, LDH, and CK, were not significantly different between the two groups (**Figures 3B–E**). Although HFD did not significantly change the FBG levels (**Figure 3F**), IVGTT at week 24 displayed significantly higher levels of blood glucose in the HFD group at most time points during the first 30 min after glucose administration, indicating an insulin-resistant state (**Figure 3G**).

### 3.3 HFD Induced Profound Perturbation of Plasma and Urine Metabolome in Dogs

**Figure 4:**
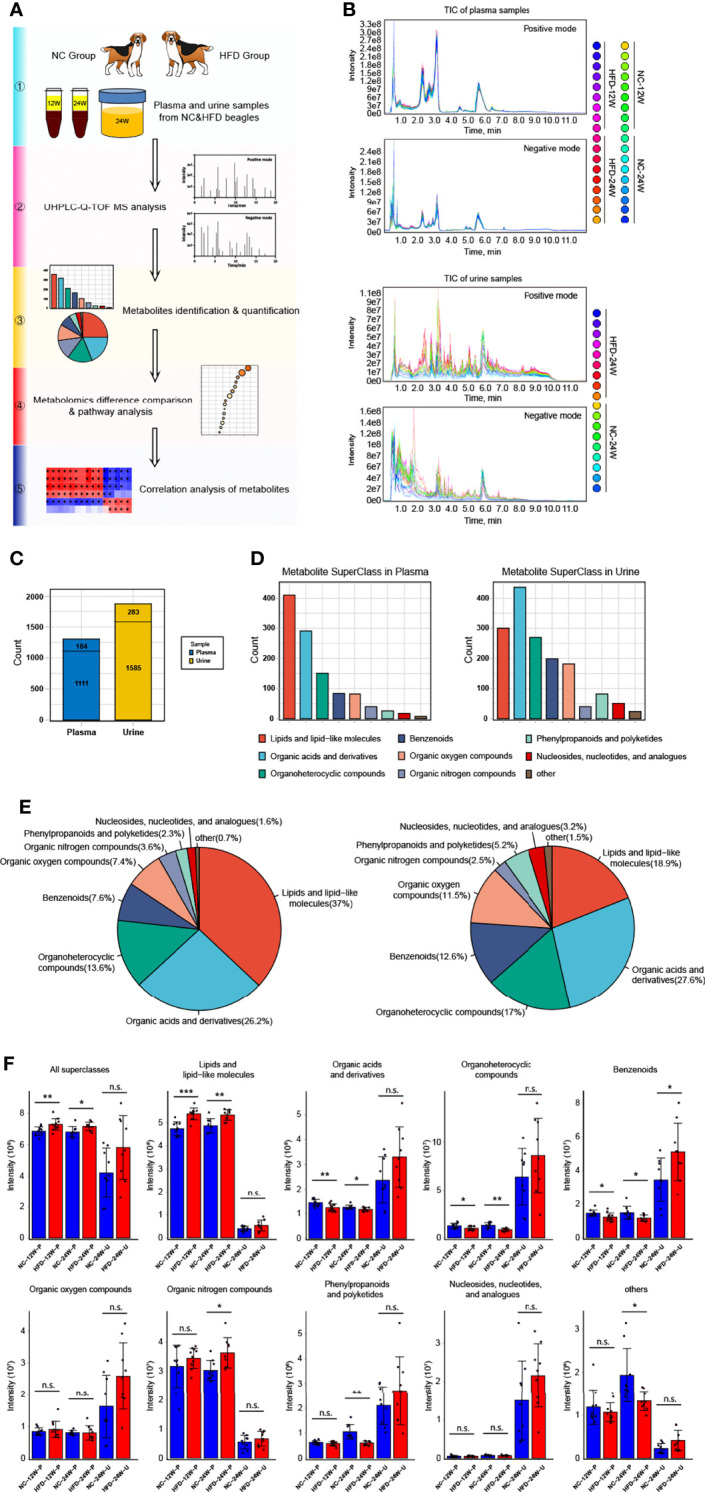
Untargeted metabolomics analysis of canine plasma and urine samples relying on UHPLC-Q-TOF MS. **(A)** Scheme for the complete process of metabolite identification, classification, difference and pathway analysis, and correlation study of 12-week plasma as well as 24-week plasma and urine of NC and HFD dogs. n = 9 per group. **(B)** Plasma (top) and urine (bottom) samples overlapped with the total ion chromatogram (TIC) of quality control samples in positive and negative ion modes, respectively. **(C)** The total number of metabolites with or without the attribution information of chemical taxonomy detected in plasma and urine samples. **(D, E)** Statistics of all identified metabolites according to the attribution information of chemical taxonomy. The specific number **(D)** and the proportion **(E)** of each metabolite superclass in plasma (left) and urine (right) samples are presented, respectively. **(F)** Relative intensity of the mass spectrometry signal of each metabolite superclass identified in plasma and urine samples from the two groups. n = 9 per group. Data are expressed as the mean ± SD. n.s. represents not statistically significant. Significance levels *P < 0.05, **P < 0.01, ***P < 0.001.

To obtain deep insight into the metabolic characteristics of the HFD canine model, we performed nontargeted metabolomic analysis based on blood and urine samples from HFD-fed and NC-fed dogs collected at week 12 and week 24 (**Figures 4A, B**). A total of 1295 metabolites were detected in the plasma, 1111 of which had chemical taxonomy attribution information. Urine metabolomic analysis identified 1868 compounds, among which 1585 had their classification information (**Figure 4C**). Among the detected plasma metabolites with chemical taxonomy information, 37.0% were lipids and lipid-like molecules, followed by organic acids and derivatives (26.2%) and organoheterocyclic compounds (13.6%). For the detected compounds in urine, 27.6% were organic acids and derivatives, followed by lipids and lipid-like molecules (18.9%) and organoheterocyclic compounds (17.0%) (**Figures 4D, E**). The abundance of each superclass of the detected metabolites in the plasma and urine samples for both groups is shown in **Figure 4F**. The names, chemical taxonomy attribution and other information of all detected metabolites are detailed in the **Supplementary Table 1**.

#### 3.3.1 HFD Induced Profound Perturbation of Plasma Metabolome

**Figure 5:**
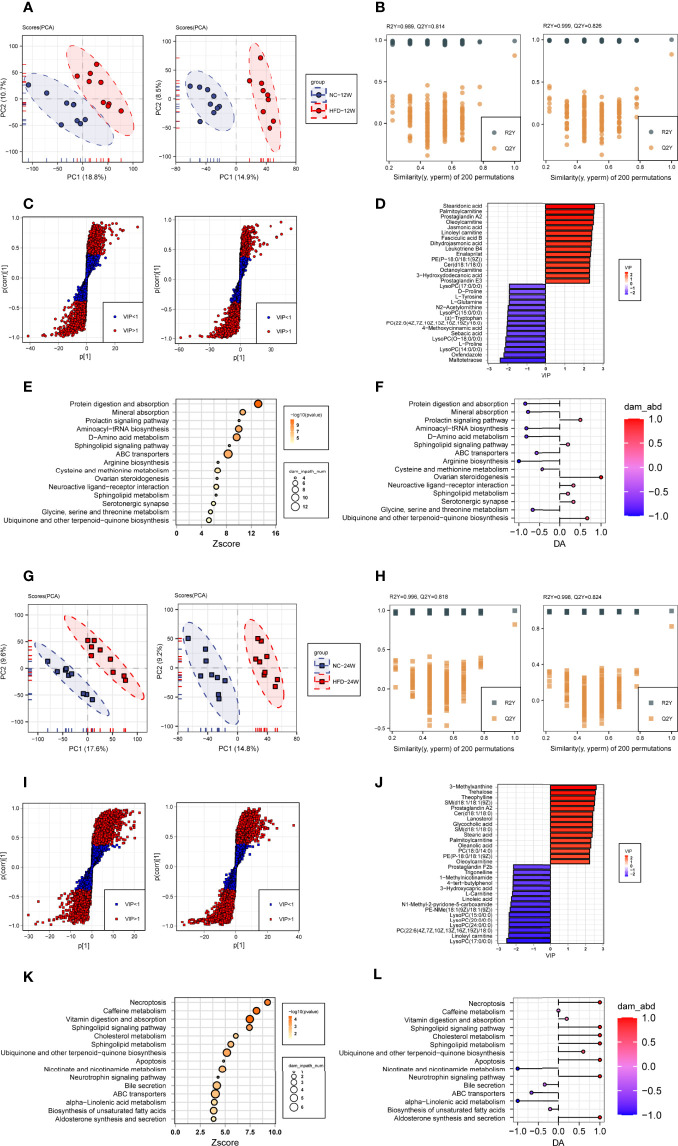
Plasma metabolome changes related to HFD-induced metabolic disorder. **(A, B)** Principal component analysis (**A**, PCA) and orthogonal partial least squares discriminant analysis (**B**, OPLS-DA) of canine plasma samples at 12 weeks in positive (left) and negative (right) ion modes. **(C)** Screening of differential metabolites based on the variable importance for the projection (VIP) in the OPLS-DA model in positive (left) and negative (right) ion modes. Significant difference was defined as VIP > 1 and *P* < 0.05. **(D)** The endogenous differential metabolites with the highest fold change of expression after merging positive and negative ion patterns. **(E, F)** Kyoto Encyclopedia of Genes and Genomes (KEGG) pathway enrichment analysis of 153 differential metabolites in 12-week plasma **(E)** and differential abundance (DA) score of these pathways **(F)**. **(G–L)** Metabonomics analysis and metabolite screening of canine plasma samples at 24 weeks corresponding to **(A–F)**.

We compared the levels of individual metabolites in the plasma of NC-fed and HFD-fed dogs at week 12 and week 24. At week 12, PCA showed clear separation of the two groups based on both the negative and positive metabolites (**Figure 5A**). PC1 and PC2 explained 29.5% of the total variation in the levels of the positive metabolites and 23.4% of that in the levels of the negative metabolites. We further conducted OPLS-DA (orthogonal partial least squares-discriminant analysis) to discriminate between the nonobese and obese samples and found the metabolites that mostly contributed to the discrimination (**Figure 5B**). OPLS-DA of the positive metabolites showed that R2Y equals 0.989 and Q2 equals 0.814, and OPLS-DA of the negative metabolites showed an R2Y of 0.999 and Q2 of 0.826, indicating high-quality discrimination.

In total, 153 plasma metabolites (69 positive and 84 negative) with significantly different levels between the NC and HFD groups were identified (VIP > 1, *P* < 0.05) at 12 weeks, with 89 significantly higher and 64 significantly lower relative concentration metabolites in the HFD-fed dogs (**Figure 5C**). Among the endogenous metabolites with elevated levels in the HFD group, stearidonic acid had the largest VIP, followed by palmitoylcarnitine, prostaglandin A2, oleoylcarnitine, and jasmonic acid. In contrast, among the endogenous metabolites with decreased concentrations in the HFD group, maltotetraose had the largest VIP, followed by oxfendazole, lysoPC(14:0/0:0), L-proline, and lysoPC(O-18:0/0:0) (**Figure 5D**). Pathway analysis showed that differential metabolites were mainly engaged in processes such as protein digestion and absorption, mineral absorption, the prolactin signaling pathway, aminoacyl-tRNA biosynthesis, D-amino acid metabolism, and the sphingolipid signaling pathway (**Figures 5E, F**).

At week 24, separations were evidenced by the two groups in PCA of both the negative and positive plasma metabolites (**Figure 5G**). The OPLS-DA model also indicated high-quality discrimination (R2Y = 0.996, Q2 = 0.818 in the positive mode; R2Y = 0.998, Q2 = 0.824 in the negative mode) (**Figure 5H**). We identified 175 plasma metabolites (94 negative and 81 positive) that varied between the NC and HFD groups (VIP > 1, *P* < 0.05) (**Figure 5I**). Among the 80 metabolites with increased abundance in obese dogs, those endogenous with the top VIP included 3-methylxanthine, trehalose, theophylline, SM(d18:1/18:1(9Z)), prostaglandin A2, and Cer(d18:1/18:0). Among the 95 metabolites with decreased abundance in the HFD group, those endogenous with the top VIP were mainly phosphocholine, acylcarnitines, and fatty acids (**Figure 5J**). Pathway analysis revealed that the differential metabolites mainly participate in processes such as necroptosis, caffeine metabolism, vitamin digestion and absorption, sphingolipid signaling pathway, and cholesterol metabolism (**Figures 5K, L**).

**Figure 6:**
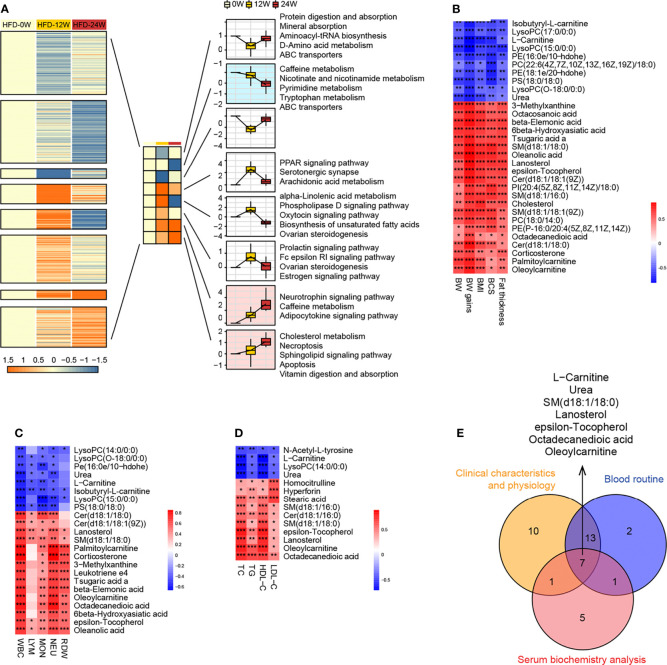
Correlation research between plasma metabolites and animal phenotypes representing obesity and metabolic disorder. **(A)** Dynamic pattern diagram of KEGG pathway variation during 0, 12 and 24 weeks of HFD feeding. All pathways are divided into eight clusters according to the trend characteristics of K-means at three time points. Clusters with the trend of continuously declining (mint blue) or elevated (light pink) K-means were selected for subsequent analysis. **(B–D)** Heatmaps indicate the correlation between plasma endogenous metabolites and animal phenotypes related to metabolic disorders, including physical examination **(B)**, routine blood tests **(C)**, and serum biochemistry **(D)**. Significance levels *P < 0.05, **P < 0.01, ***P < 0.001. **(E)** Venn diagram shows 7 endogenous metabolites that are significantly associated with all three phenotypes.

To investigate which pathways were consistently affected by HFD during the feeding period, we analyzed the dynamic patterns of pathways at three time points, i.e., weeks 0, 12, and 24 (**Figure 6A** **)**. The pathways with a continuously increasing trend in K-means across the three time points included the neurotrophin signaling pathway, caffeine metabolism, adipocytokine signaling pathway, cholesterol metabolism, necroptosis, sphingolipid signaling pathway, apoptosis, and vitamin digestion and absorption, whereas the pathways with a continuously decreasing trend in K-means included caffeine metabolism, nicotinate and nicotinamide metabolism, pyrimidine metabolism, tryptophan metabolism, and ABC transporters, suggesting that HFD could affect these pathways in a time-related manner.

To further identify the plasma metabolites most correlated with the phenotypes, we performed correlation analysis between individual metabolites and physical examination parameters (**Figure 6B**), complete blood count parameters (**Figure 6C**), and blood lipid indicators (**Figure 6D**). Similar to the differential metabolites identified between the HFD-fed and NC-fed groups, the metabolites most correlated with those phenotypes were primarily lipids. A Venn diagram showed that 7 metabolites, including L-carnitine, urea, epsilon-tocopherol, octadecanedioic acid, SM(d18:1/18:0), lanosterol, and oleoylcarnitine, were significantly correlated with all three phenotypes (**Figure 6E**).

#### 3.3.2 HFD Induced the Profound Perturbation of Urine Metabolome

**Figure 7:**
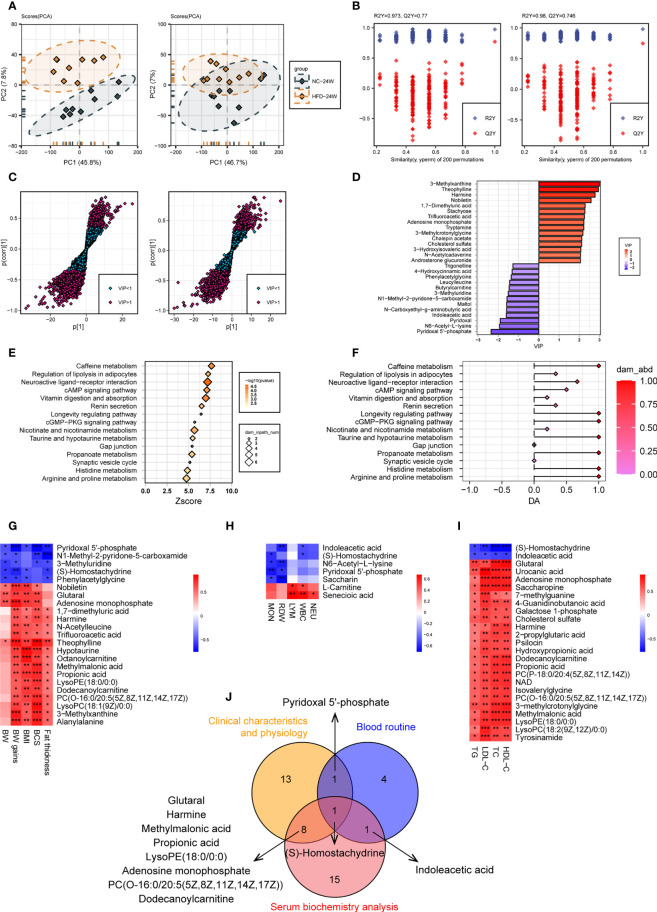
Urine metabolome features related to HFD-induced metabolic disorder. **(A, B)** PCA **(A)** and OPLS-DA **(B)** of canine urine samples at 24 weeks in positive (left) and negative (right) ion modes, respectively. **(C)** Screening of differential metabolites based on VIP values in the OPLS-DA model in positive (left) and negative (right) ion modes. Significant difference was defined as VIP > 1 and *P* < 0.05. **(D)** The endogenous differential metabolites with the highest fold change of expression after merging positive and negative ion patterns. **(E–F)** KEGG pathway enrichment analysis of 132 differential metabolites in 24-week urine **(E)** and DA score of these pathways **(F)**. **(G–I)** Heatmaps demonstrate the correlation between urine endogenous metabolites and canine phenotypes connected with metabolic disorders, including physical examination **(G)**, routine blood tests **(H)**, and serum biochemistry **(I)**. Significance levels *P < 0.05, **P < 0.01, ***P < 0.001. **(J)** Venn diagram for metabolites that were significantly associated with multiple phenotypes.

We compared the levels of individual metabolites in the urine of NC-fed and HFD-fed dogs at week 24. A PCA of the positive metabolites displayed clear separation of the two groups, wherein PC1 and PC2 explained 53.6% of the total variation; however, separations were less evident in the PCA of the negative metabolites (**Figure 7A**). OPLS-DA of both positive and negative metabolites indicated high-quality discrimination (R2Y = 0.973, Q2 = 0.770 in the positive mode; R2Y = 0.980, Q2 = 0.746 in the negative mode) (**Figure 7B**).

A total of 132 urine metabolites (46 negative and 86 positive) with significantly different levels between the two groups were identified (VIP > 1, *P* < 0.05) (**Figure 7C**). Among the 103 metabolites with higher levels in the HFD group, those endogenous with the top VIP included 3-methylxanthine, theophylline, harmine, nobiletin, and 1,7-dimethyluric acid. Among the 29 metabolites with lower levels in the HFD group, those endogenous with the top VIP included pyridoxal 5’-phosphate, N6-acetyl-L-lysine, pyridoxal, and indoleacetic acid (**Figure 7D**). Pathway analysis showed that the differential metabolites were mainly engaged in processes such as caffeine metabolism, regulation of lipolysis in adipocytes, neuroactive ligand-receptor interaction, cAMP signaling pathway, and vitamin digestion and absorption (**Figures 7E, F**). **Figures 7G–I** presents heatmaps displaying the correlation between endogenous metabolites and the three phenotypes. A Venn diagram showed that S-homostachydrine was significantly correlated with all three phenotypes, and metabolites such as pyridoxal 5’-phosphate, harmine, propionic acid, adenosine monophosphate, and dodecanoylcarnitine were significantly correlated with two of the phenotypes (**Figure 7J**). Details of the differential metabolites identified from plasma or urine are presented in the **Supplementary Table 2**.

#### 3.3.3 Combined Plasma-Urine Metabolome Analysis

**Figure 8:**
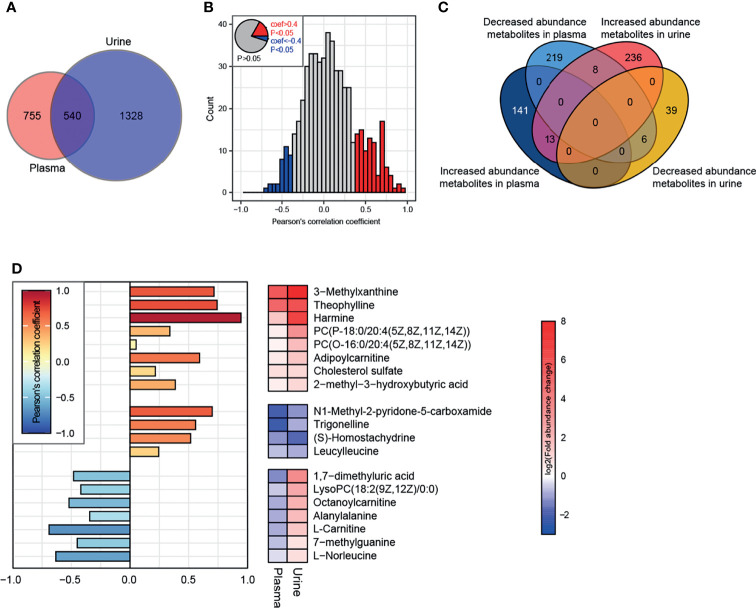
Conjoint analysis of plasma-urine metabolomics. **(A)** A total of 1295 and 1868 metabolites (containing endogenous and exogenous metabolites and including those without attribution information of chemical taxonomy) were identified in plasma and urine, respectively. **(B)** Correlation analysis of metabolites in plasma and urine. Metabolites satisfying *P* < 0.05 and Pearson’s correlation coefficient > 0.4 or < −0.4 are marked as red and blue, respectively. **(C)** Characteristics of abundance variation in plasma/urine of all metabolites from **(B)**. **(D)** The specific characteristics of abundance variation in each body fluid of 19 endogenous metabolites extracted from the intersection are shown in **(C)**. There were 8 exogenous metabolites whose characteristics are not illustrated.

There were 540 metabolites detected in both the plasma and urine (**Figure 8A**). Correlation analysis indicated that the metabolites with a significant correlation in abundance in blood and urine represented only a small proportion of the total metabolites (**Figure 8B**). Further analysis identified eight metabolites, such as 3-methylxanthine, theophylline, harmine, adipoylcarnitine, and cholesterol sulfate, that had increased abundance in both the plasma and urine of the HFD-fed dogs, while 4 metabolites had decreased abundance in both the plasma and urine, including N1-methyl-2-pyridone-5-carboxamide, trigonelline, S-homostachydrine, and leucylleucine (**Figures 8C, D**). Interestingly, the abundance of L-carnitine and octanoylcarnitine was decreased in the plasma but increased in the urine of HFD-fed dogs.

## 4 Discussion

Canine models are increasingly used in metabolic studies due to their physiological similarity with humans. In the present study, we established a canine model of obesity and metabolic disorders induced by HFD. The phenotype was evidenced by imaging examinations and repeated measurements of body weight, BCS, and serum biochemistry during the feeding period. Dogs fed a HFD for 24 weeks were obese and had impaired insulin sensitivity and elevated serum lipid levels and inflammatory cell counts. Thus, this HFD-induced canine model would serve as an appropriate tool to study obesity-related metabolic disorders in the laboratory setting.

The metabolome characteristics of obese dogs in the present study showed similarity to those in human studies. The metabolites most associated with human obesity are primarily lipids and those involved in lipid metabolism (e.g., fatty acids, acylcarnitines, and phospholipids), in addition to branched-chain and aromatic amino acids and metabolites involved in nucleotide metabolism (26–28). Similarly, most differential plasma metabolites in this study were lipids such as fatty acids and metabolites involved in fatty acid biosynthesis and oxidation and sphingolipid metabolism. The marked changes in plasma lipids in both human and canine obesity indicate that altered energy substrate metabolism occurs in an obese state (21, 29). It is noteworthy that the metabolome profiles of rodent models of HFD-induced obesity, which are widely used to study human metabolic disorders, vary greatly among different studies. For instance, while some studies with HFD-fed rodent models displayed a metabolome pattern similar to that of obese humans, where differential metabolites are mainly involved in lipid and energetic metabolism (30, 31), others showed distinctly different patterns where differential metabolites are mainly engaged in processes such as amino acid metabolism (e.g., tryptophan metabolism, phenylalanine and tyrosine metabolism), gut microbiota metabolism and insulin resistance-related metabolism (26, 32).

Previous human metabolomics studies found higher levels of short- and medium-chain acylcarnitines in obese compared to lean individuals, which might result from impaired fatty acid biosynthesis and oxidation (33). In addition, a canine study showed that several acylcarnitines were increased in the plasma of dogs undergoing acute weight gain (34). In agreement with these findings, our study identified higher levels of palmitoylcarnitine, oleoylcarnitine, linoleylcarnitine, octanoylcarnitine, dodecanoylcarnitine, and adipoylcarnitine in the plasma or urine of HFD-fed dogs than in NC dogs. However, whether the changes in carnitine concentration are the cause or consequence of HFD-induced metabolic disorders requires further investigation.

Several fatty acids, such as linolenic acid and stearidonic acid, were identified as differential metabolites in this study. Linolenic acid is an essential fatty acid that belongs to the omega-3 fatty acid group. It is highly concentrated in certain plant oils and has been shown to be beneficial against obesity and its related pathologies in both human subjects and animal models (35–39). Human metabolomics studies revealed that the serum concentrations of linoleic acid were altered among the obese groups after consuming a standardized high-calorie meal (40). Several randomized controlled trials (RCTs) have been conducted to analyze the effect of linolenic acid and its derived metabolites as a dietary treatment on obesity-related conditions. Ando et al. reported that repeated consumption of α-linolenic acid-enriched diacylglycerol enhances postprandial fat metabolism after a meal, which is related to its visceral fat area-reducing effect (38, 41). Another RCT with 59 hypertriglyceridemic participants showed that foods supplemented with vegetable oils rich in linoleic acid or α-linolenic acid could effectively decrease the levels of total cholesterol and low-density-lipoprotein cholesterol (42). These results suggest linolenic acid as a dietary supplement for treating obesity and its related metabolic disorders.

Vitamin deficiency is common in human obesity and metabolic disorders (43). In the present study, vitamin E and pyridoxal 5’-phosphate were significantly associated with metabolic phenotypes. Obesity-related metabolic disorders are associated with augmented oxidative stress, and vitamin E has the most significant antioxidant potential among fat-soluble vitamins. Human studies have confirmed an association between vitamin E deficiency and increased abdominal adiposity in obese individuals (44–46). Pyridoxal 5’-phosphate is a coenzyme of many enzymatic reactions and is the active form of vitamin B6. It has been shown to inhibit acetyl-CoA carboxylase isoforms (47). Moreover, plasma pyridoxal 5’-phosphate levels positively correlated with the fat-free mass percentage in human subjects (48). Thus, vitamin E and pyridoxal 5’-phosphate may serve as potential therapeutic targets for treating obesity and metabolic disorders.

Our analysis identified 8 metabolites with increased abundance in both plasma and urine in HFD-fed dogs, such as 3-methylxanthine, theophylline, harmine, and adipoylcarnitine, and 4 metabolites with decreased abundance in both plasma and urine, such as trigonelline. Trigonelline is a gut flora-derived metabolite and has been demonstrated to have antidiabetic and antiobesity effects. It can attenuate adipocyte differentiation and lipid accumulation and induce browning in white adipocytes (49, 50). In diabetic rats, the administration of trigonelline exerts insulin sensitization by attenuating oxidative stress and endoplasmic reticulum stress in the pancreas and activating PPARγ in adipose tissue (51). Harmine is a natural compound that has been shown to induce β-cell proliferation, exert an insulin-sensitizing effect, and induce adipocyte thermogenesis in animal models, improving systemic metabolism (52, 53). Thus, trigonelline and harmine are promising compounds for the treatment of obesity and metabolic disorders. An interesting finding in the present study is that several metabolites related to caffeine metabolism, such as 3-methylxanthine and theophylline, were increased in HFD-fed dogs. Dietary caffeine has been shown to have antiobesity effects (36). *In vitro* experiments found that caffeine and its metabolites, such as theophylline and 3-methylxanthine, inhibit insulin-stimulated glucose uptake in differentiated adipocytes and suppress intracellular lipid accumulation (37). Moreover, methylxanthine derivative-rich cacao extracts can decrease fat accumulation in adipocytes by inhibiting adipocyte differentiation (38). Several RCTs indicated that caffeine intake, in combination with ephedra, glucosyl hesperidin, or high-intensity interval training, causes weight loss and improves metabolic risk factors in obese subjects (54–56). These results suggest an antiobesity and metabolically improving capacity of caffeine and its metabolites. Further investigation is required to investigate the role of other metabolites in obesity and metabolic disorders and their translational potentials.

## Conclusions

In the present study, we established a canine model of obesity and metabolic disorders induced by HFD in the laboratory setting and found profound perturbation of the metabolome of HFD-fed dogs. Several compounds, such as linolenic acid, vitamin E, pyridoxal 5’-phosphate, trigonelline, harmine, 3-methylxanthine, and theophylline, may serve as potential targets for the prevention and treatment of obesity and metabolic disorders. However, further validations in clinical trials are needed before they can be implemented in clinics.

## Data Availability Statement

The original contributions presented in the study are included in the article/**Supplementary Material**. Further inquiries can be directed to the corresponding authors.

## Ethics Statement

The animal study was reviewed and approved by the Animal Care and Use Committee of Renmin Hospital of Wuhan University (No.WDRM 20201219C, approved 28 December 2020).

## Author Contributions

WQ, ZC, TZ, YFH, X-JZ, and XC conceived and designed the methods, experiments, analysis, and interpretation. XH, YPH, and YZ, collection and preparation of samples. WQ, ZC, XH, ST, JW, RL, JX, MH, and JZ, data collection and conduction of experiments. WQ, YPH, YFH, and XC performed the analysis of obtained data. ZC, X-JZ, Z-GS, and HL, project supervision, and contribution to the interpretation of the results. WQ, ZC, XH, and XC, writing the manuscript, and figure preparation. LB, YD, and XZ contributed to the critical revision of the article. All authors have read and authorized the submitted version of the manuscript.

## Funding

This work was supported by the National Science Foundation of China (Grant No. 81770053, 81970364, 82000538 and 82100086), the Hubei Province Innovation Platform Construction Project (Grant No. 20204201117303072238), the Henan Charity General Federation-Hepatobiliary Foundation of Henan Charity General Federation (Grant No. GDXZ2019010) and the Wuhan Science and Technology Planning Project (Grant No. 2020021105012439).

## Conflict of Interest

The authors declare that the research was conducted in the absence of any commercial or financial relationships that could be construed as a potential conflict of interest.

## Publisher’s Note

All claims expressed in this article are solely those of the authors and do not necessarily represent those of their affiliated organizations, or those of the publisher, the editors and the reviewers. Any product that may be evaluated in this article, or claim that may be made by its manufacturer, is not guaranteed or endorsed by the publisher.
